# Commentary on physicians’ satisfaction with providing buprenorphine treatment by Knudsen et al.

**DOI:** 10.1186/s13722-019-0164-2

**Published:** 2019-08-26

**Authors:** Gavin Bart, Jeffrey Samet

**Affiliations:** 1 Hennepin Healthcare, 701 Park Avenue, Minneapolis, MN 55415 USA; 2 Boston University Schools of Medicine and Public Health and Boston Medical Center, 801 Massachusetts Avenue, Boston, MA 02118 USA

**Keywords:** Buprenorphine, Professional satisfaction, Primary care, Opioid use disorder

Knudsen et al. report findings of physician satisfaction with providing buprenorphine treatment across three states [1]. While this report of baseline data from a larger study evaluating different approaches to implementing buprenorphine treatment for opioid use disorder (OUD) into ambulatory settings [2] is small in sample size and reach, it raises an important issue that should be addressed for all projects attempting to expand the number of providers offering buprenorphine: provider satisfaction.

Since the approval of buprenorphine in 2002, there have been extensive efforts to increase the number of physicians obtaining buprenorphine waivers under the Drug Addiction Treatment Act of 2000 (DATA 2000). These efforts include peer mentoring through the Providers’ Clinical Support System, learning collaboratives such as the Extension for Community Healthcare Outcomes (Project ECHO), dissemination of national practice guidelines (e.g., by the Veterans Affairs/Department of Defense and the American Society of Addiction Medicine), and numerous live and web-based continuing medical education events. In 2016, the Comprehensive Addiction and Recovery Act allowed certain advanced practice providers to also become DATA-waived. Because of these efforts, more than 65,000 providers are now DATA-waived. Despite these efforts, less than two-thirds of these DATA-waived providers actually prescribe buprenorphine [3]. Several studies have attempted to understand the discrepancy between the number of waivered providers and the number of providers who actually prescribe buprenorphine [4–6]. Not prescribing buprenorphine—or prescribing below the authorized patient limit of 30, 100, or 275—is often described as resulting from lack of time, poor system support, insufficient training, poor reimbursement, and lack of behavioral health support, amongst others. Knudsen et al. are the first to focus on professional satisfaction as a moderating factor to the expansion of buprenorphine into general medical settings.

While Knudsen et al. found that overall professional satisfaction did not differ between DATA-waived physicians and non-waived physicians who were involved in the care of patients with OUD, they did find that among the buprenorphine physicians, satisfaction specific to the buprenorphine scope of care was lower than their overall professional satisfaction. Further, dissatisfaction in buprenorphine practice was related to DATA-waiver patient limits. Physicians with a 100 patient limit were more dissatisfied than those with either a 30 or 275 patient limit. Domains specifically identified include frustration with buprenorphine work and failure of buprenorphine work to meet expectations. These findings stand in contrast to the few qualitative reports describing the empowerment that some primary care physicians feel in treating patients with buprenorphine.

The interaction between professional satisfaction with buprenorphine and the model of practice may be critical in differentiating why some providers are satisfied and others dissatisfied with buprenorphine practice. Since overall professional satisfaction did not differ between the DATA-waived and non-waived physicians in Knudsen, et al., these findings suggest that it is not just the practice model overall but specifically how that model adopts buprenorphine that may be important. Many delivery models for buprenorphine have been described and it may be that a particular setting may find one model to be more acceptable than another [7]. Clearly, identifying practice setting types and characteristics of those practices will be key to a better understanding of physician and other providers’ satisfaction with buprenorphine prescribing. While Knudsen et al. did not include practice model or non-physician buprenorphine prescribers in their analysis, there may be a clue within their data. It is of interest that providers with a 100 patient limit were more dissatisfied than those with either the 30 or 275 patient limit. Presumably it takes little practice support to add fewer than 30 buprenorphine patients; to add up to 275 may require specific practice changes. Providers in the middle ground of 100 patients may not have achieved enough practice change to allow them to provide care in a satisfying manner. Only further study will delineate the interactions between practice management, provider satisfaction, and, ultimately, quality of care.

Given that a number of models of buprenorphine delivery in medical settings are occurring as reviewed by Korthuis et al. [7], research specifically assessing physician and other providers’ satisfaction with providing this care within these models merits examination. One may surmise that active uptake and efforts to replicate one specific model, the Massachusetts Collaborative Care Model [8], is an indicator of provider satisfaction, but given the confluence of motivations for expanding treatment, that conclusion merits confirmation.

The risks of ignoring physician and other waivered providers’ satisfaction may be high. In general, professional satisfaction and physician burnout are increasingly recognized as significantly influencing the business and practice of medicine [9]. Patient treatment adherence and patient satisfaction may be inversely related to physician professional satisfaction [10]. Burnout-related provider turnover is estimated to cost a health system $7600 per employed provider per year and has been shown to negatively affect patient satisfaction, if not health outcomes [11]. For buprenorphine specifically, provider loss will leave patients at risk as they may not be able to immediately connect with another DATA-waived provider. Adding professional satisfaction metrics to buprenorphine workforce expansion offers new opportunities for study and intervention. The outcomes of such satisfaction assessments should impact the decision as to what model is adopted by a clinic or health system growing this kind of enterprise. As we promote expanded integration of buprenorphine into general medical settings, for our patients and for our colleagues, we must be prepared to address the following question for buprenorphine prescribers: *Are you satisfied*?

## Data Availability

Not applicable.
